# Expression of a bacterial 3-dehydroshikimate dehydratase (QsuB) reduces lignin and improves biomass saccharification efficiency in switchgrass (*Panicum virgatum* L.)

**DOI:** 10.1186/s12870-021-02842-9

**Published:** 2021-01-21

**Authors:** Zhangying Hao, Sasha Yogiswara, Tong Wei, Veronica Teixeira Benites, Anagh Sinha, George Wang, Edward E. K. Baidoo, Pamela C. Ronald, Henrik V. Scheller, Dominique Loqué, Aymerick Eudes

**Affiliations:** 1 Joint BioEnergy Institute, Emeryville, CA 94608 USA; 2 Environmental Genomics and Systems Biology Division, Lawrence Berkeley National Laboratory, Berkeley, CA 94720 USA; 3 Department of Plant Pathology and the Genome Center, University of California, Davis, CA 95616 USA; 4 Present address: State Key Laboratory of Agricultural Genomics, BGI-Shenzhen, Shenzhen, 518000 China; 5 Biological Systems and Engineering Division, Lawrence Berkeley National Laboratory, Berkeley, CA 94720 USA; 6 Department of Plant and Microbial Biology, University of California-Berkeley, Berkeley, CA 94720 USA

**Keywords:** Switchgrass, Lignin, Shikimate, Protocatechuate, Saccharification, Bioenergy

## Abstract

**Background:**

Lignin deposited in plant cell walls negatively affects biomass conversion into advanced bioproducts. There is therefore a strong interest in developing bioenergy crops with reduced lignin content or altered lignin structures. Another desired trait for bioenergy crops is the ability to accumulate novel bioproducts, which would enhance the development of economically sustainable biorefineries. As previously demonstrated in the model plant Arabidopsis, expression of a 3-dehydroshikimate dehydratase in plants offers the potential for decreasing lignin content and overproducing a value-added metabolic coproduct (i.e., protocatechuate) suitable for biological upgrading.

**Results:**

The 3-dehydroshikimate dehydratase QsuB from *Corynebacterium glutamicum* was expressed in the bioenergy crop switchgrass (*Panicum virgatum* L.) using the stem-specific promoter of an O-methyltransferase gene (*pShOMT*) from sugarcane. The activity of *pShOMT* was validated in switchgrass after observation in-situ of beta-glucuronidase (GUS) activity in stem nodes of plants carrying a *pShOMT::GUS* fusion construct. Under controlled growth conditions, engineered switchgrass lines containing a *pShOMT::QsuB* construct showed reductions of lignin content, improvements of biomass saccharification efficiency, and accumulated higher amount of protocatechuate compared to control plants. Attempts to generate transgenic switchgrass lines carrying the QsuB gene under the control of the constitutive promoter *pZmUbi-1* were unsuccessful, suggesting possible toxicity issues associated with ectopic QsuB expression during the plant regeneration process.

**Conclusion:**

This study validates the transfer of the QsuB engineering approach from a model plant to switchgrass. We have demonstrated altered expression of two important traits: lignin content and accumulation of a co-product. We found that the choice of promoter to drive *QsuB* expression should be carefully considered when deploying this strategy to other bioenergy crops. Field-testing of engineered QsuB switchgrass are in progress to assess the performance of the introduced traits and agronomic performances of the transgenic plants.

**Supplementary Information:**

The online version contains supplementary material available at 10.1186/s12870-021-02842-9.

## Background

The development of biorefineries to reduce our dependence on nonrenewable fossil fuel resources requires production of dedicated bioenergy crops that can be grown with few inputs on marginal lands. Other desired traits for bioenergy crops include high biomass yields, stress resilience, reduced recalcitrance to conversion into biofuels and bioproducts, and the accumulation of valuable co-products [1, 2]. Switchgrass has long been recognized as an ideal crop for bioenergy purposes considering its pest and disease resistance, high biomass yields, growth performance on poor soils due to relatively low requirements for added fertilizers, carbon sequestration capacity via its extensive root system, drought tolerance, and efficient water use [3]. As a consequence, significant efforts have been implemented for the improvement of switchgrass via breeding and genetic transformation [4, 5].

**Fig. 1:**
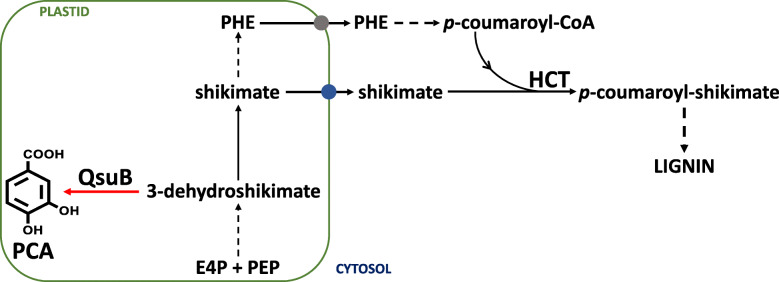
Schematic diagram of lignin biosynthesis and the conversion of 3-dehydroshikimate into protocatchuate (PCA) catalyzed by plastid-targeted QsuB. Grey and blue circles indicate a phenylalanine transporter and a putative shikimate transporter, respectively. Dashed arrows represent multiple enzymatic steps. E4P: Erythrose 4-phosphate; HCT: hydroxycinnamoyl CoA: shikimate hydroxycinnamoyl transferase; PEP: Phosphoenolpyruvate; PHE: Phenylalanine

Lignin is a major polymer in plant biomass that negatively impacts the conversion of cell wall polysaccharides into advanced bioproducts, and several engineering approaches have been established to modify lignin content and its monomeric composition [6, 7]. For example, the heterologous expression of a bacterial 3-dehydroshikimate dehydratase (QsuB) targeted to plastids resulted in strong lignin reductions (up to 50%) in Arabidopsis [8]. One explanation for this observation is the possible reduction of the cytosolic shikimate pool needed for the synthesis of *p*-coumaroyl-shikimate catalyzed by hydroxycinnamoyl CoA: shikimate hydroxycinnamoyl transferase (HCT) during lignin biosynthesis (Fig. 1).

In switchgrass, several *HCT* gene candidates have been proposed to have a role in lignin biosynthesis based on the HCT activity measured with the corresponding recombinant enzymes and their expression profile in lignifying cell suspension cultures [9, 10]. In fact, more than 90% reduction in transcript levels of either *PvHCT1* or *PvHCT2* had no effect on lignin content, but simultaneous downregulation of both genes resulted in slight decreases of lignin content (5–8%) based on the yield of lignin monomers released after thioacidolysis [11]. These results not only indicate a role for HCT in lignin biosynthesis in switchgrass, with PvHCT1 and PvHCT2 being redundant, but also suggest the involvement of additional HCTs with similar functions.

In this work, we report on the expression of QsuB in switchgrass using the promoter of a sugarcane O-methyltransferase gene (*pShOMT*) [12]. Several switchgrass QsuB transformation events show reduction of lignin content and decreased cell wall recalcitrance. A significant increase in the content of protocatechuate accumulated in biomass was also observed.

## Results

### Molecular characterization of the *pShOMT::QsuB* switchgrass lines

**Fig. 2:**
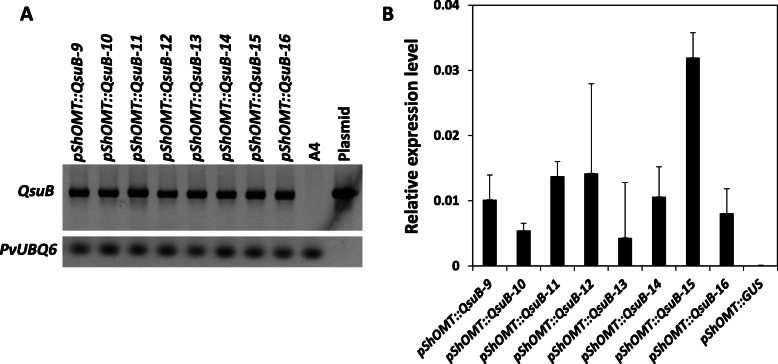
Molecular characterization of eight independent switchgrass lines containing the *pShOMT::QsuB* construct. **a** Detection of the QsuB gene by PCR. ‘A4’ is a gDNA sample from wild-type switchgrass and ‘Plasmid’ is the *pShOMT::QsuB* construct used for plant transformation. **b** Detection of QsuB transcripts by RT-qPCR. *QsuB* expression levels relative to that of *PvUBQ6* are shown. cDNA obtained from a line containing the *pShOMT::GUS* construct were used as negative control. Values are means ±SD of two biological replicates (*n* = 2)

A total of eight independent transformation events were regenerated after *Agrobacterium*-mediated transformation of switchgrass using a DNA construct that contains the plastid-targeted QsuB coding sequence fused downstream of the *pShOMT* promoter. The *QsuB* transgene was detected by PCR using gDNA from each transformant (Fig. 2a), and *QsuB* expression was validated by qPCR performed on cDNA synthesized from RNAs obtained from the first internode of each line at the E2 stage (Fig. 2b). A DNA construct consisting of *pShOMT* fused upstream of the GUS reporter gene was also transferred to switchgrass. Analysis of internodes and nodes from switchgrass plants harboring the *pShOMT::GUS* construct at the E4 stage suggested that *pShOMT* is mainly active in the nodes, whereas little activity was observed in the internodes (Figure S1). Under controlled growth conditions, all transgenic lines did not show any particular phenotype nor growth defect and were visually indistinguishable from each other or compared to non-transformed wild-type plants.

### Protocatechuate content in *pShOMT::QsuB* switchgrass

**Fig. 3:**
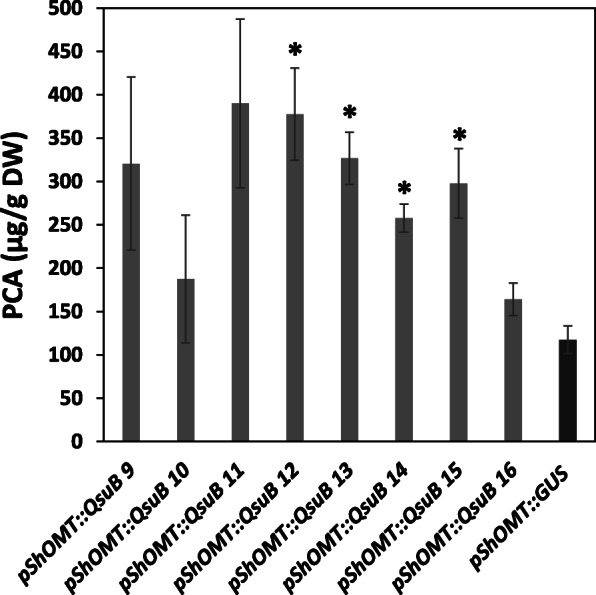
Protocatechuate (PCA) content measured in the biomass of switchgrass *pShOMT::QsuB* transgenic lines. Values are means ±SE of three biological replicates (*n* = 3). Asterisks indicate significant differences from a line containing the *pShOMT::GUS* construct using the unpaired Student’s t-test (*P < 0.05)

Protocatechuate (PCA), the product of QsuB activity, was extracted from the total aboveground biomass of switchgrass plants at the E5 stage and quantified. Compared to control plants carrying the *pShOMT::GUS* construct, PCA was significantly increased by ~ 2–3-fold in four independent *pShOMT::QsuB* lines, reaching up to 380 μg/g dry weight (Fig. 3). This data shows that expression of plastid-targeted QsuB in transgenic switchgrass enabled the conversion of endogenous 3-dehydroshikimate into PCA.

### Lignin content and biomass saccharification efficiency in *pShOMT::QsuB* switchgrass

**Fig. 4:**
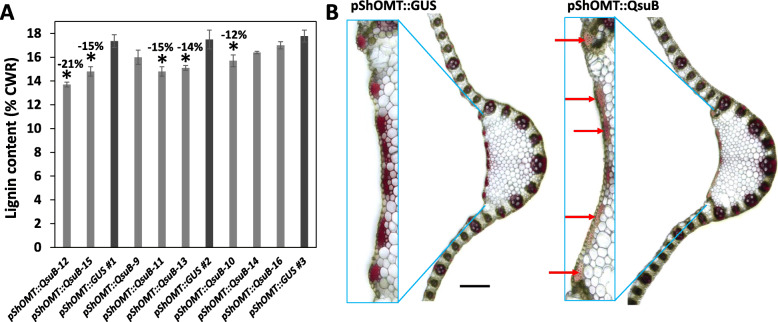
**a** Klason lignin content measured in cell wall residues (CWR) obtained from biomass of switchgrass lines containing the *pShOMT::QsuB* construct. A line containing the *pShOMT::GUS* construct was used as control and analyzed thrice since measurements were carried out in three separate batches. Values are means ±SE of four biological replicates (*n* = 4). Asterisks indicate significant differences from the line containing the *pShOMT::GUS* construct using the unpaired Student’s t-test (*P < 0.05). **b** Representative pictures of leaf blade cross-sections stained with phloroglucinol-HCl from lines containing either the *pShOMT::GUS* or the *pShOMT::QsuB* construct. Note the reduction of the staining specifically in thick fibers located in the leaf abaxial zone for the *pShOMT::QsuB* line (red arrows). Scale: black bar = 200 μm

Total lignin content in the biomass from the *pShOMT::QsuB* switchgrass lines was measured using the Klason method. Compared to control lines containing the *pShOMT::GUS* construct, several *pShOMT::QsuB* lines showed significant reductions of lignin content ranging from 12 to 21% (Fig. 4a). Inspection of stem sections treated with phloroglucinol-HCl for the staining of lignin did not reveal any differences between the different *pShOMT::QsuB* lines and the control *pShOMT::GUS* lines (data not shown). However, on leaf blade sections, reductions in the intensity of the typical red staining were observed in the case of the *pShOMT::QsuB* lines compared to controls, especially in thick fibers located in the abaxial zone (Fig. 4b).

**Fig. 5:**
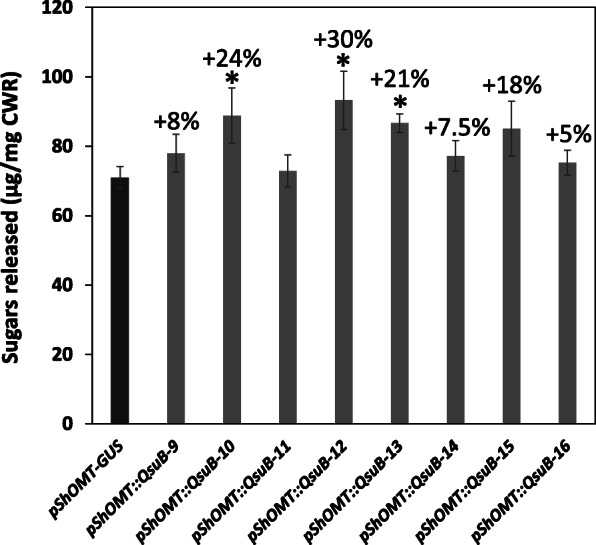
Saccharification of cell wall residues (CWR) obtained from biomass of switchgrass lines containing the *pShOMT::QsuB* construct. A line containing the *pShOMT::GUS* construct was used as control. Amounts of sugars released from CWR after a hot water pretreatment and 72 h of enzymatic digestion with cellulase are shown. Values are means ±SE of four biological replicates (*n* = 4). Asterisks indicate significant differences from the control using the unpaired Student’s t-test (*P < 0.05)

The recalcitrance towards enzymatic degradation of the biomass of the engineered switchgrass was evaluated by measuring the amount of sugars released from cell wall residues after pretreatment with hot water followed by a 72-h hydrolysis using a commercial cellulase cocktail (CTec2). As shown in Fig. 5, higher amount of reducing sugars was obtained for several *pShOMT::QsuB* lines compared to the *pShOMT::GUS* control lines, with significant increases ranging between 21 and 30%.

## Discussion

Here, we describe the successful expression of the bacterial 3-dehydroshikimate dehydratase QsuB gene under the control of *pShOMT* in switchgrass. We show that the resulting plants display 12–21% reduction in lignin, a 2–3-fold increase in the bioaccumulation of PCA and a 5–30% increase in saccharification efficiency.

*pShOMT* was previously shown to be preferentially active in stem vascular tissues in sugarcane, rice, maize, and sorghum [12], making it a good promoter candidate to express *QsuB* specifically in lignifying tissues within vascular bundles. Similar to previous observations made in sugarcane, we were able to detect GUS activity in stem nodes from switchgrass lines carrying a *pShOMT::GUS* construct. Nevertheless, an apparent reduction of lignin content observed in some discrete regions of leaf blades (i.e., fibers on the adaxial zone) from plants carrying the *pShOMT::QsuB* construct indicate that *pShOMT* is also active in leaf cells with secondary wall accumulation (Fig. 4b). In addition to *pShOMT*, attempts to generate transgenic switchgrass lines with constructs containing *QsuB* under the control of the constitutive promoter of the maize ubiquitin1 gene (*pZmUbi-1*) was unsuccessful, whereas only a single event was obtained with a *pZmCesa10::QsuB* construct containing the promoter of the maize cellulose synthase gene *CESA10* involved in secondary cell wall formation [13] (Figures S2, S3). This is possibly the result of toxicity occurring during the plant regeneration process when using these two *pZmUbi-1::QsuB* and *pZmCesa10::QsuB* constructs. Considering that QsuB diverts lignin biosynthesis, using the promoter of a lignin biosynthetic gene to drive *QsuB* expression may be more suited spatial-temporally during plant development. Interestingly, the single *pZmCesa10::QsuB* line showed a reduction of total lignin content as well as reduced phloroglucinol staining in leaf fibers (Figure S2E, F). Obtaining more switchgrass transgenic events with the *pZmCesa10::QsuB* construct will be essential to validate the effectiveness of *pZmCesa10* in driving QsuB expression to reduce lignin content.

The exact mechanism by which QsuB expression reduces lignin in switchgrass is still unresolved; in particular, whether the cytosolic pools of shikimate —required for HCT activity— and *p*-coumaroyl-shikimate are reduced remain to be demonstrated. Similarly, it would be interesting to determine the lignin monomeric composition in the different QsuB switchgrass lines, especially the relative amount of *p*-hydroxyphenyl (H) units, which is known to be higher in Arabidopsis QsuB plants and typically increases in HCT down-regulated dicot species [8, 14–20]. Furthermore, the recent discovery in several plant species —including switchgrass— of genes encoding putative 3-hydroxylases (C3H) that convert *p*-coumarate to caffeate, as well as genetic evidence of their role in lignin formation in *Brachypodium distachyon*, question the exclusive role of HCT and the involvement of *p*-coumarate esters during lignin biosynthesis in monocots [21].

The overproduction of PCA in switchgrass lines expressing QsuB probably results from a partial conversion of the endogenous pool of 3-dehydroshikimate catalyzed by QsuB activity. Notably, increases in PCA titers (2–3-fold compared to control switchgrass) are smaller than those previously reported in Arabidopsis and tobacco plants containing the QsuB gene under the control of the promoter of the Arabidopsis cinnamate 4-hydroxylase gene (*pAtC4H*), which were at least two orders of magnitude higher compared to controls plants [8, 22]. In connection with these observations, it has been demonstrated in vitro that PCA acts as a competitive inhibitor of at least one HCT isoform from switchgrass (i.e., PvHCT2) [23]. Therefore, it would be informative to attempt to identify putative *p*-coumaroyl-protocatechuate conjugates in metabolite extracts from *pShOMT::QsuB* switchgrass to determine if such HCT promiscuous activity —and possibly HCT inhibition— also occurs in vivo. Finally, it is promising to observe that the QsuB engineering strategy has the potential to enhance PCA titers in switchgrass biomass because several techno-economic analyses demonstrated the benefits of producing co-products *in planta* to render bioenergy crops economically sustainable [1, 24, 25]. In fact, several studies have already reported on the use of PCA as carbon source or pathway intermediate for the biological synthesis of diverse valuable products such as beta-ketoadipic acid, muconolactone, muconic acid, 2-pyrone-4,6-dicarboxylic acid, bisabolene, and methyl ketones [22, 26–30].

## Conclusion

The QsuB engineering approach has been established in switchgrass. This work highlights the fact that selecting an adequate promoter to drive *QsuB* expression should be an important parameter for successful engineering of other crops with this gene via tissue culture-dependent transformation methods. Considering that *pShOMT* activity is induced in the leaf and root by key regulators of biotic and abiotic stress responses such as salicylic acid, jasmonic acid and methyl jasmonate [12], it will be essential to field test our engineered *pShOMT::QsuB* switchgrass to assess its agronomic performance and resilience to environmental stress.

## Methods

### Vector construction and plant transformation

The promoters *pShOMT* [12], *pZmCesa10* (2.6 kb located upstream the start codon of the maize CESA10 gene - GenBank: AY372244.1), and *pZmUbi-1* [31] were synthesized with the following flanking restriction sites: 5′-AscI / 3′-AvrII for *pShOMT* and 5′-HindIII / 3′-AvrII for *pZmCesa10* and *pZmUbi-1* (Genscript, Piscataway, NJ). Promoter sequences were released by enzyme digest and ligated into the binary vector pA6-GW [32] pre-digested with either AscI/AvrII or HindII/AvrII to generate respectively the *pA6-pShOMT-GW*, *pZmCesa10-GW*, and *pA6-pZmUbi-1-GW* binary vectors. The entry vector pDONR221-schl::QsuB containing the gene encoding the 3-dehydroshikimate dehydratase QsuB from *Corynebacterium glutamicum* preceded with the nucleotide sequence of a chloroplast transit peptide [8] was LR recombined with the *pA6-pShOMT-GW*, *pA6-pZmCesa10-GW*, and *pA6-pZmUbi-1-GW* vectors using the Gateway cloning technology (Thermo Fisher Scientific, Waltham, MA) to generate the constructs *pA6-pShOMT-schl::QsuB*, *pA6-pZmCesa10-GW-schl::QsuB*, and *pA6-pZmUbi-1-GW-schl::QsuB*, respectively. A nucleotide sequence encoding the beta-glucuronidase gene (GUS) from *E. coli* was amplified from pCAMBIA1301 using primers flanked with attB1 (5′) and attB2 (3′) Gateway recombination sites, and inserted into the *pA6-pShOMT-GW* and *pA6-pZmCesa10-GW* vectors by Gateway cloning to generate the constructs *pA6-pShOMT::GUS* and *pA6*-*pZmCesa10::GUS*, respectively. Cloning primers are listed in Table S1. The binary vectors were transformed into *Agrobacterium tumefaciens* strain AGL1 for switchgrass (*Panicum virgatum* L.,) transformation which was performed at the University of Missouri’s Plant Transformation Core Facility as previously described [33], where embryogenic calli used for transformation were induced from mature seeds of switchgrass cultivar Alamo-A4 (Hancock Farm & Seed Company, Dade City, FL). Hygromycin B (Life Technologies, Foster City, CA) was added to the selection medium at 50 mg/L.

### Plant growth conditions

Four transgenic switchgrass plants for each event were transferred to 2-gal pots containing Pro-Mix soil and grown in a room at 22 °C and 60% humidity using a light intensity of 250 μmol/m^2^/s and 16 h of light per day.

### PCR genotyping

Genomic DNA was extracted from leaf tissue obtained from one of the clones from each event using the Plant DNeasy plant mini kit (Qiagen, Carlsbad, CA). PCR primers specific to the *QsuB* gene were used to detect the transgene, and primers specific to the switchgrass *PvUBQ6* gene (GenBank: FE609298.1) were used to assess the quality of the gDNA. All the primers used in this study are listed in Table S1.

### RT-qPCR

Total RNAs were extracted from the first internode collected from plants at the E2 stage [34] using the TRIzol reagent (Thermo Fisher Scientific, Waltham, MA) and cDNA synthesis was conducted using the high-capacity cDNA reverse transcription kit (Applied BioSystems, Foster City, CA) as previously described [35]. RT-qPCR was performed as described previously using 40 cycles consisting of 5 s at 95 °C for denaturation and 15 s at 60 °C for annealing and amplification [35]. The relative quantification of *QsuB* transcripts was calculated using the 2-^Δ^CT method and normalized to the reference gene *PvUBQ6* (GenBank: FE609298.1). The results are the average from two biological replicates which were each analyzed in technical replicates. RT-qPCR primers are listed in Table S1.

### Lignin assays

The Wiesner histochemical test using phloroglucinol-HCl, a reagent that reacts with coniferaldehyde groups in lignin, was performed on transverse sections of stems and leaf blades from plants at the E2 stage as previously described [36, 37]. For Klason lignin measurements, whole switchgrass plants were cut at the E5 stage (no visible flag leaf) 3 cm from the bottom, and biomass was dried in an oven at 50 °C for 7 days. Dried biomass was grinded with a Model 4 Wiley Mill equipped with a 1-mm mesh (Thomas Scientific, Swedesboro, NJ). Grinded biomass was extracted as previously described [8] and Klason lignin was measured using the standard NREL biomass protocol [38].

### Saccharification assays

Grinded and extracted biomass obtained from plants at the E5 stage was ball-milled to a fine powder using a Mixer Mill MM 400 (Retsch Inc., Newtown, PA) and stainless-steel balls. For saccharification assays, four biological replicates of 10 mg of fine biomass powder from each line was pretreated with liquid hot water followed by a 72-h enzymatic hydrolysis using 1% w/w Cellic CTec2 enzyme mixture (Novozymes, Denmark) as previously described [35]. Hydrolysates were used for measurement of reducing sugars using the 3,5-dinitrosalicylic acid (DNS) assay [39].

### Protocatechuate measurements

Whole switchgrass plants were cut 3 cm from the bottom at the E5 stage (no visible flag leaf), and biomass was dried in an oven at 50 °C for 7 days. Dried biomass was grinded with a Model 4 Wiley Mill equipped with a 1-mm mesh (Thomas Scientific, Swedesboro, NJ). An aliquot of the grinded biomass was ball-milled to a fine powder using a Mixer Mill MM 400 (Retsch Inc., Newtown, PA) and stainless-steel balls. Metabolites were extracted from 200 mg of dried ball-milled biomass using 80% (v/v) methanol:water followed by an acid hydrolysis step as previously described [8]. Protocatechuate was detected in metabolite extracts using high-performance liquid chromatography (HPLC), electrospray ionization (ESI), and time-of-flight (TOF) mass spectrometry (MS) as previously described [40]. Quantification was performed using a six-point calibration curve from protocatechuate solutions prepared with an authentic standard (Sigma-Aldrich, St. Louis, MO).

### Histochemical GUS assays

Stem and leaf sections were obtained manually from plants at the E4 stage using a razor blade. GUS assays were conducted on plant sections using 2 mM 5-bromo-4-chloro-3-indolyl-β-D-glucuronide (Sigma-Aldrich, St. Louis, MO) as substrate for 48 h at 37 °C as previously described [41]. After incubation, sections were dehydrated in 95% (v/v) ethanol prior to observation of the GUS staining in 70% (v/v) ethanol.

## Supplementary Information

**Additional file 1: Figure S1.** Representative pictures showing GUS activities in various tiller sections of switchgrass lines harboring the *pShOMT::GUS* construct. GUS expression is specifically observed in stem nodes. Scale: White bars = 2 mm, black bar = 400 *μ* m. N: node; IN: internode, IS: internode transverse section.

**Additional file 2: Figure S2.** Characterization of a switchgrass line harboring the *pZmCesa10::QsuB* construct. **(A)** Representative pictures showing GUS activities in various tiller sections of switchgrass lines harboring the *pZmCesa10::GUS* construct. GUS expression is mostly observed in internodes, especially in developing vascular bundles (red arrows). Scale: White bars = 2 mm, black bar = 400 *μ* m. N: node; IN: internode, IS: internode transverse section. **(B)** Detection of the *QsuB* gene by PCR in line *pZmCesa10::QsuB-5*. **(C)** Detection of *QsuB* transcripts by RT-qPCR. *QsuB* expression levels relative to that of *PvUBQ6* are shown. Values are means ±SD of two biological replicates (*n* = 2). **(D)** Protocatechuate (PCA) content measured in the biomass of the switchgrass line *pZmCesa10::QsuB-5*. A line containing the *pZmCesa10::GUS* construct was used as control. Values are means ±SE of three biological replicates (*n* = 3). Asterisks indicate a significant difference from the control using the unpaired Student’s t-test (*P < 0.001). **(E)** Klason lignin content measured in cell wall residues (CWR) obtained from the biomass of the switchgrass line *pZmCesa10::QsuB-5*. A line containing the *pZmCesa10::GUS* construct was used as control. Values are means ±SE of four biological replicates (*n* = 4). Asterisks indicate a significant difference from the control using the unpaired Student’s t-test (*P < 0.05). **(F)** Representative pictures of stem and leaf blade cross-sections stained with phloroglucinol-HCl from line *pZmCesa10::QsuB-5* and a line containing the *pZmCesa10::GUS* construct. Note in the leaves the reduction of the staining specifically in thick fibers located in both the adaxial and abaxial zones for the line *pZmCesa10::QsuB-5* (red arrows).

**Additional file 3: Table S1.** Oligonucleotides used in the study.

**Additional file 4: Figure S3.** Full length unprocessed images of PCR gels used for Figs. 2a and S2B. Note that the seven transformants obtained with the *pZmUbi-1::QsuB* construct were all false positives and did not contain the QsuB gene (see purple rectangle on the PCR gel).

## Data Availability

The authors ensure the availability of supporting data and materials. The datasets used and/or analysed during the current study are available from the corresponding author on reasonable request.

## Abbreviations

CWR: Cell wall residue
GUS: Beta-glucuronidase
HCT: hydroxycinnamoyl-CoA: shikimate hydroxycinnamoyl transferase
HPLC-ESI-TOF-MS: High-performance liquid chromatography electrospray ionization and time-of-flight mass spectrometry
PCA: Protocatechuate
RT-qPCR: Real-time quantitative reverse transcription PCR
