# Supplementary material for: Expression of a bacterial 3-dehydroshikimate dehydratase (QsuB) reduces lignin and improves biomass saccharification efficiency in switchgrass (Panicum virgatum L.)
Source: BMC Plant Biol. 2021 Jan 21;21:56. doi: 10.1186/s12870-021-02842-9 (PMC7819203; doi:10.1186/s12870-021-02842-9)
Supplement: Supplementary file 1 — Additional file 1: Figure S1. Representative pictures showing GUS activities in various tiller sections of switchgrass lines harboring the pShOMT::GUS construct. GUS expression is specifically observed in stem nodes. Scale: White bars = 2 mm, black bar = 400 μ m. N: node; IN: internode, IS: internode transverse section. [file 12870_2021_2842_MOESM1_ESM.pdf]

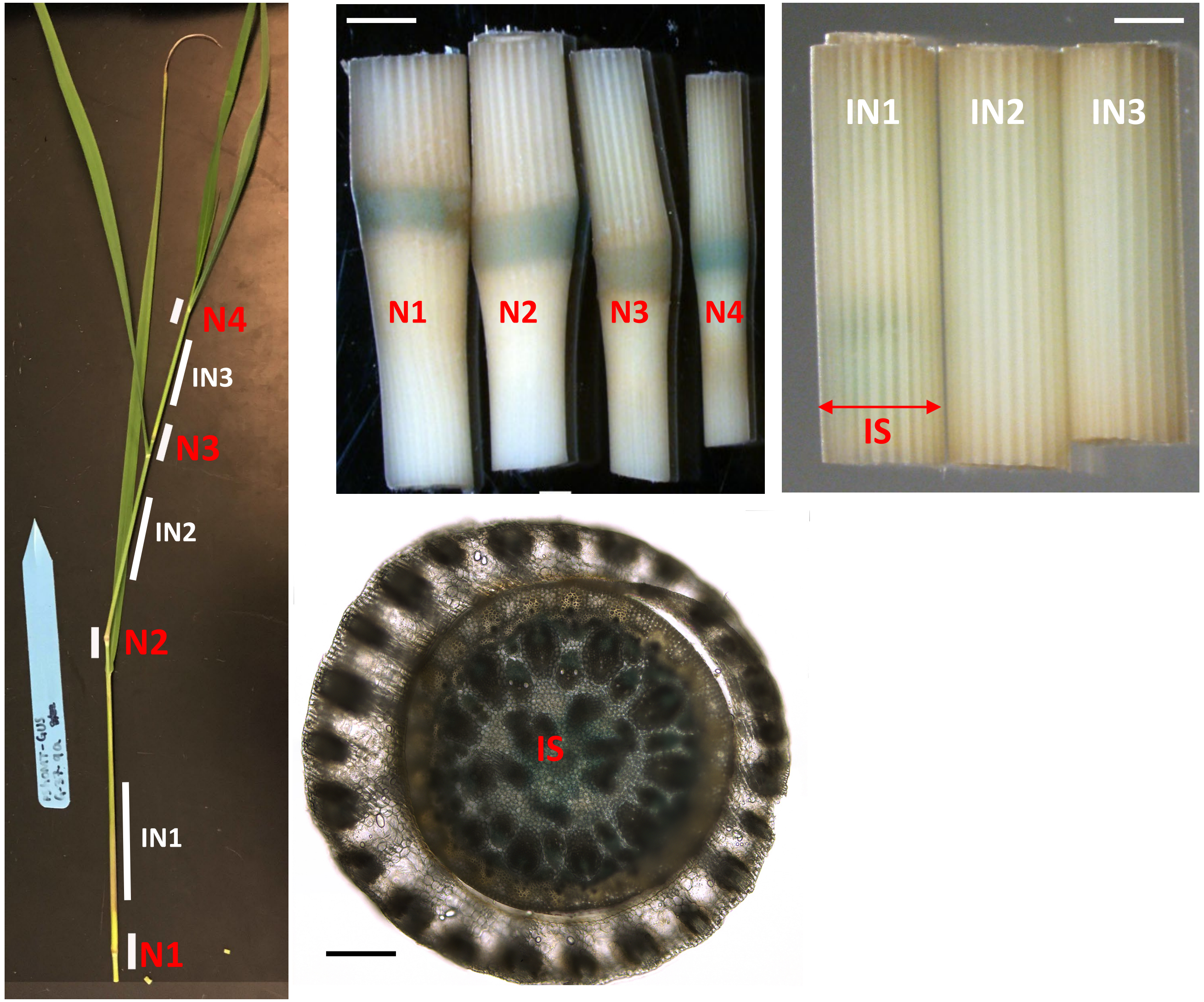

Figure S1: Representative pictures showing GUS activities in various tiller sections of switchgrass lines harboring the *pShOMT::GUS* construct. GUS expression is specifically observed in stem nodes. Scale: White bars = 2 mm, black bar = 400 µm. N: node; IN: internode, IS: internode transverse section.
