# Supplementary material for: Expression of a bacterial 3-dehydroshikimate dehydratase (QsuB) reduces lignin and improves biomass saccharification efficiency in switchgrass (Panicum virgatum L.)
Source: BMC Plant Biol. 2021 Jan 21;21:56. doi: 10.1186/s12870-021-02842-9 (PMC7819203; doi:10.1186/s12870-021-02842-9)
Supplement: Supplementary file 2 — Additional file 2: Figure S2. Characterization of a switchgrass line harboring the pZmCesa10::QsuB construct. (A) Representative pictures showing GUS activities in various tiller sections of switchgrass lines harboring the pZmCesa10::GUS construct. GUS expression is mostly observed in internodes, especially in developing vascular bundles (red arrows). Scale: White bars = 2 mm, black bar = 400 μ m. N: node; IN: internode, IS: internode transverse section. (B) Detection of the QsuB gene by PCR in line pZmCesa10::QsuB-5. (C) Detection of QsuB transcripts by RT-qPCR. QsuB expression levels relative to that of PvUBQ6 are shown. Values are means ±SD of two biological replicates (n = 2). (D) Protocatechuate (PCA) content measured in the biomass of the switchgrass line pZmCesa10::QsuB-5. A line containing the pZmCesa10::GUS construct was used as control. Values are means ±SE of three biological replicates (n = 3). Asterisks indicate a significant difference from the control using the unpaired Student’s t-test (*P < 0.001). (E) Klason lignin content measured in cell wall residues (CWR) obtained from the biomass of the switchgrass line pZmCesa10::QsuB-5. A line containing the pZmCesa10::GUS construct was used as control. Values are means ±SE of four biological replicates (n = 4). Asterisks indicate a significant difference from the control using the unpaired Student’s t-test (*P < 0.05). (F) Representative pictures of stem and leaf blade cross-sections stained with phloroglucinol-HCl from line pZmCesa10::QsuB-5 and a line containing the pZmCesa10::GUS construct. Note in the leaves the reduction of the staining specifically in thick fibers located in both the adaxial and abaxial zones for the line pZmCesa10::QsuB-5 (red arrows). [file 12870_2021_2842_MOESM2_ESM.pdf]

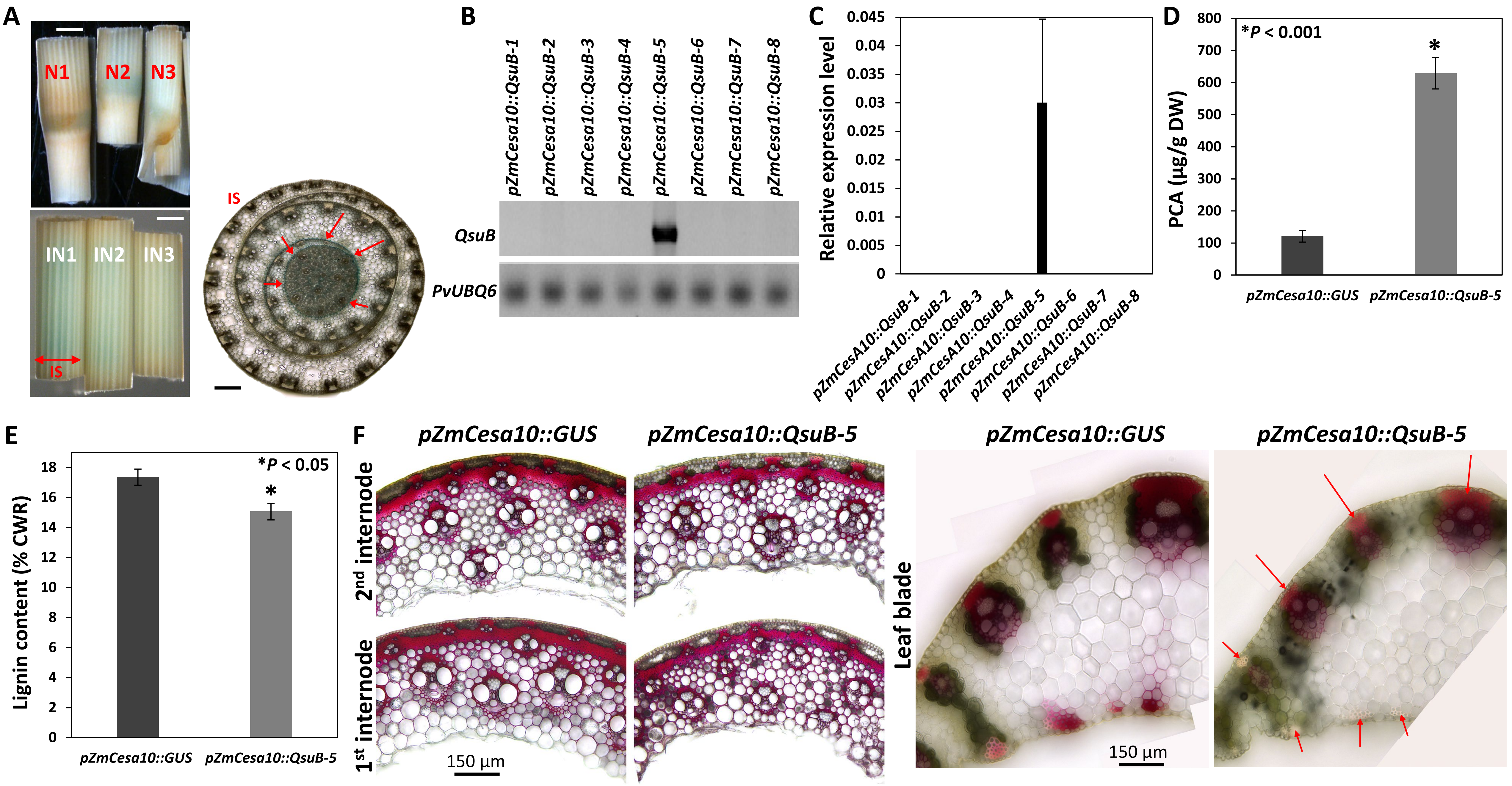
A
N1
N2
N3
IN1
IN2
IN3
IS
IS
B
pZmCesa10::QsuB-1
pZmCesa10::QsuB-2
pZmCesa10::QsuB-3
pZmCesa10::QsuB-4
pZmCesa10::QsuB-5
pZmCesa10::QsuB-6
pZmCesa10::QsuB-7
pZmCesa10::QsuB-8
QsuB
PvUBQ6
C
Relative expression level
0.045
0.04
0.035
0.03
0.025
0.02
0.015
0.01
0.005
0
pZmCesA10::QsuB-1
pZmCesA10::QsuB-2
pZmCesA10::QsuB-3
pZmCesA10::QsuB-4
pZmCesA10::QsuB-5
pZmCesA10::QsuB-6
pZmCesA10::QsuB-7
pZmCesA10::QsuB-8
D
*P < 0.001
PCA (µg/g DW)
800
700
600
500
400
300
200
100
0
*
pZmCesa10::GUS
pZmCesa10::QsuB-5
E
Lignin content (% CWR)
*P < 0.05
18
16
14
12
10
8
6
4
2
0
*
pZmCesa10::GUS
pZmCesa10::QsuB-5
F
pZmCesa10::GUS
pZmCesa10::QsuB-5
pZmCesa10::GUS
pZmCesa10::QsuB-5
2nd internode
1st internode
Leaf blade
150 µm
150 µm

Figure S2: Characterization of a switchgrass line harboring the *pZmCesa10::QsuB* construct. **(A)** Representative pictures showing GUS activities in various tiller sections of switchgrass lines harboring the *pZmCesa10::GUS* construct. GUS expression is specifically observed in internodes, especially in developing vascular bundles (red arrows). Scale: White bars = 2 mm, black bar = 400 µm. N: node; IN: internode, IS: internode transverse section. (**B**) Detection of the *QsuB* gene by PCR in line *pZmCesa10::QsuB-5*. (**C**) Detection of *QsuB* transcripts by RT-qPCR. *QsuB* expression levels relative to that of *PvUBQ6* are shown. Values are means ±SD of two biological replicates (n = 2). **(D)** Protocatechuate (PCA) content measured in the biomass of the switchgrass line *pZmCesa10::QsuB-5*. A line containing the *pZmCesa10::GUS* construct was used as control. Values are means ±SE of three biological replicates (n = 3). Asterisks indicate a significant difference from the control using the unpaired Student's t-test (*$P < 0.001$). **(E)** Klason lignin content measured in cell wall residues (CWR) obtained from the biomass of the switchgrass line *pZmCesa10::QsuB-5*. A line containing the *pZmCesa10::GUS* construct was used as control. Values are means ±SE of four biological replicates (n = 4). Asterisks indicate a significant difference from the control using the unpaired Student's t-test (*$P < 0.05$). (**F**) Representative pictures of stem and leaf blade cross-sections stained with phloroglucinol-HCl from line *pZmCesa10::QsuB-5* and a line containing the *pZmCesa10::GUS* construct. Note in the leaves the reduction of the staining specifically in thick fibers located in both the adaxial and abaxial zones for the line *pZmCesa10::QsuB-5* (red arrows).
