# Supplementary material for: Expression of a bacterial 3-dehydroshikimate dehydratase (QsuB) reduces lignin and improves biomass saccharification efficiency in switchgrass (Panicum virgatum L.)
Source: BMC Plant Biol. 2021 Jan 21;21:56. doi: 10.1186/s12870-021-02842-9 (PMC7819203; doi:10.1186/s12870-021-02842-9)
Supplement: Supplementary file 3 — Additional file 3: Table S1. Oligonucleotides used in the study. [file 12870_2021_2842_MOESM3_ESM.docx]

| **Table S1**. Oligonucleotides used in the study. | | |
| --- | --- | --- |
| **Primer name** | **Application** | **Sequence (5’-3’)** |
| GUS-attB1 | Cloning | GGGGACAAGTTTGTACAAAAAAGCAGGCTTCatggtccgtcctgtag |
| GUS-attB2 |  | GGGGACCACTTTGTACAAGAAAGCTGGGTCtcagcagcagggaggc |
| QsuB-Fw | Genotyping | TggCTTCgATCTCCTCCTCA |
| QsuB-Rv |  | gCATCAgCCAATTgCACgAA |
| PvUBQ6-Fw | RT-qPCR / genotyping | agaagcgcaagaagaagacg |
| PvUBQ6-Rv |  | ccaccttgtagaactggagca |
| QsuB-qpcr-Fw | RT-qPCR | CgCTCCTgACggAgTTgAAT |
| QsuB-qpcr-Rv |  | gTTgCCAAggCTgAgCAAAg |
