# Supplementary material for: Expression of a bacterial 3-dehydroshikimate dehydratase (QsuB) reduces lignin and improves biomass saccharification efficiency in switchgrass (Panicum virgatum L.)
Source: BMC Plant Biol. 2021 Jan 21;21:56. doi: 10.1186/s12870-021-02842-9 (PMC7819203; doi:10.1186/s12870-021-02842-9)
Supplement: Supplementary file 4 — Additional file 4: Figure S3. Full length unprocessed images of PCR gels used for Figs. 2a and S2B. Note that the seven transformants obtained with the pZmUbi-1::QsuB construct were all false positives and did not contain the QsuB gene (see purple rectangle on the PCR gel). [file 12870_2021_2842_MOESM4_ESM.pdf]

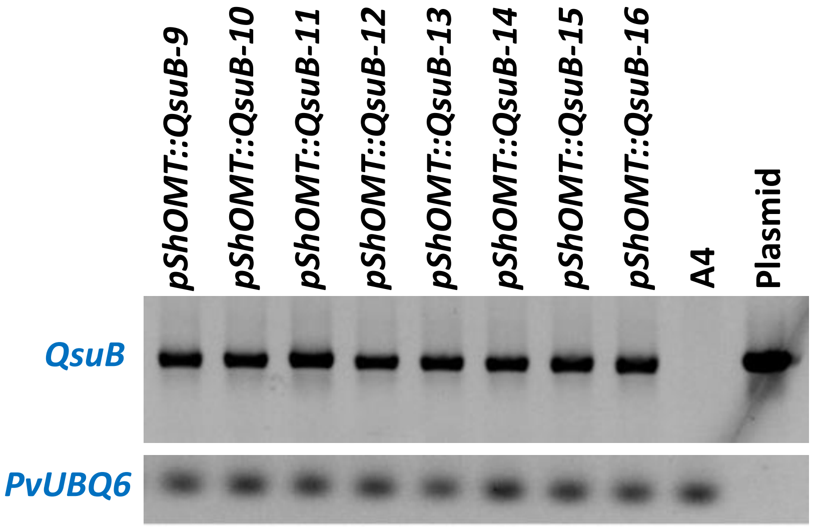

Figure 2A

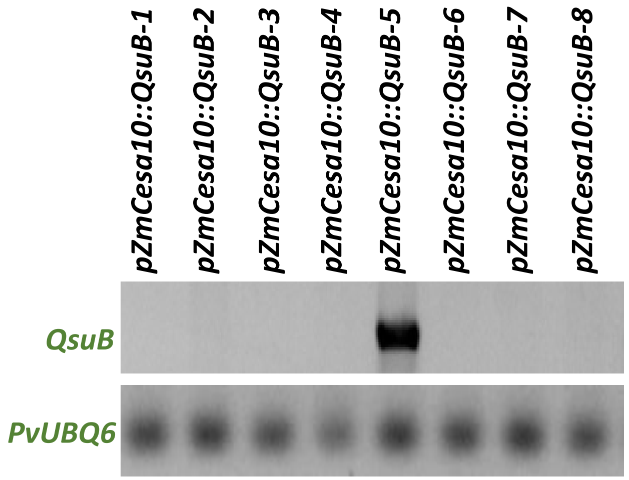

Figure S2B

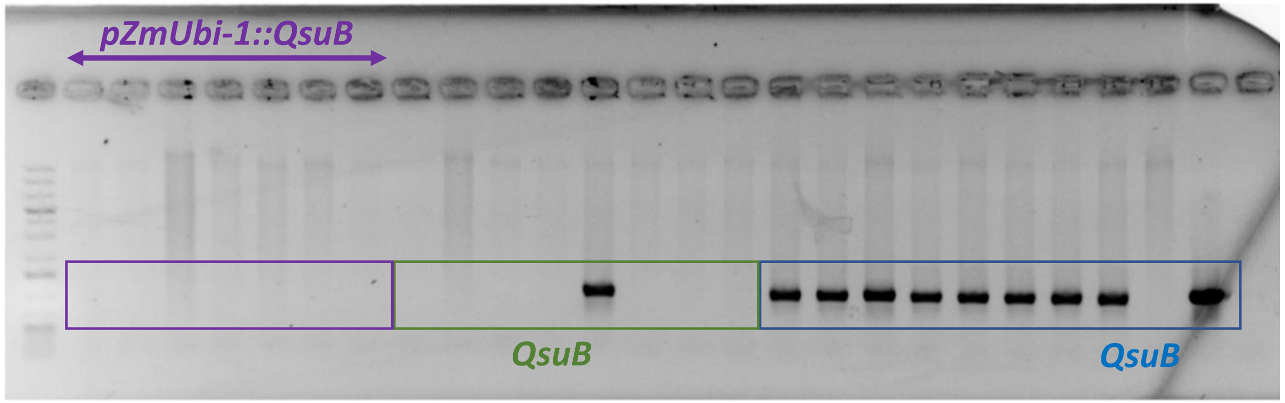

Original image for PCR with QsuB primers

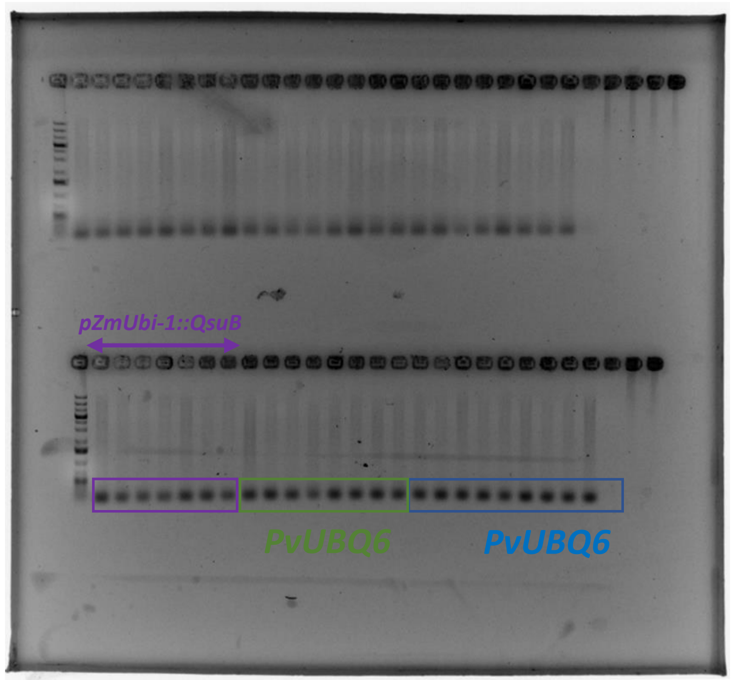

Original image for PCR with PvUBQ6 primers

Figure S3: Full length unprocessed images of PCR gels used for figures 2A and S2B. Note that the seven transformants obtained with the *pZmUbi-1::QsuB* construct were all false positives and did not contain the QsuB gene (see purple rectangle on the PCR gel).
